# Loss of HER2 and decreased T-DM1 efficacy in HER2 positive advanced breast cancer treated with dual HER2 blockade: the SePHER Study

**DOI:** 10.1186/s13046-020-01797-3

**Published:** 2020-12-10

**Authors:** Giulia Bon, Laura Pizzuti, Valentina Laquintana, Rossella Loria, Manuela Porru, Caterina Marchiò, Eriseld Krasniqi, Maddalena Barba, Marcello Maugeri-Saccà, Teresa Gamucci, Rossana Berardi, Lorenzo Livi, Corrado Ficorella, Clara Natoli, Enrico Cortesi, Daniele Generali, Nicla La Verde, Alessandra Cassano, Emilio Bria, Luca Moscetti, Andrea Michelotti, Vincenzo Adamo, Claudio Zamagni, Giuseppe Tonini, Giacomo Barchiesi, Marco Mazzotta, Daniele Marinelli, Silverio Tomao, Paolo Marchetti, Maria Rosaria Valerio, Rosanna Mirabelli, Antonio Russo, Maria Agnese Fabbri, Nicola D’Ostilio, Enzo Veltri, Domenico Corsi, Ornella Garrone, Ida Paris, Giuseppina Sarobba, Francesco Giotta, Carlo Garufi, Marina Cazzaniga, Pietro Del Medico, Mario Roselli, Giuseppe Sanguineti, Isabella Sperduti, Anna Sapino, Ruggero De Maria, Carlo Leonetti, Angelo Di Leo, Gennaro Ciliberto, Rita Falcioni, Patrizia Vici

**Affiliations:** 1 Cellular Network and Molecular Therapeutic Target Unit, IRCCS Regina Elena National Cancer Institute, Via Elio Chianesi 53, 00144 Rome, Italy; 2 Division of Medical Oncology 2, IRCCS Regina Elena National Cancer Institute, Via Elio Chianesi 53, 00144 Rome, Italy; 3 Pathology Department, IRCCS Regina Elena National CancerInstitute, Rome, Italy; 4 Area of Translational Research, IRCCS Regina Elena National Cancer Institute, Rome, Italy; 5 Department of Medical Sciences, University of Turin, Turin, Italy; 6 Candiolo Cancer Institute, FPO-IRCCS, Candiolo, Turin, Italy; 7 Medical Oncology, Sandro Pertini Hospital, Rome, Italy; 8 Oncology Clinic, “Ospedali Riuniti di Ancona” Hospital, Ancona, Italy; 9 Radiotherapy Unit, Department of Oncology, Careggi University Hospital, Florence, Italy; 10 Medical Oncology Unit, St Salvatore Hospital, L’Aquila, Italy; 11 Department of Medical, Oral and Biotechnological Sciences, University Gabriele D’Annunzio, Chieti, Italy; 12 Department of Medical Oncology, University La Sapienza, Rome, Italy; 13 Breast Cancer Unit, ASST Cremona, Cremona, Italy; 14 Oncology Unit, ASST Fatebenefratelli Sacco-PO Fatebenefratelli, Milan, Italy; 15 Oncology Unit, IRCCS Foundation Polyclinic University A. Gemelli, University Cattolica Del Sacro Cuore, Rome, Italy; 16 University of Verona, Verona, Italy; 17 Department of Oncology and Hematology, University Hospital, Modena, Italy; 18 UO Medical Oncology, S. Chiara Hospital, Pisa, Italy; 19 Medical Oncology Unit, A.O. Papardo & Department of Human Pathology, University of Messina, Messina, Italy; 20 Medical Oncology Unit, Addarii Institute of Oncology, S. Orsola-Malpighi Hospital, Bologna, Italy; 21 Department of Oncology, University Campus Biomedico, Rome, Italy; 22 Medical Oncology Unit, Sant’Andrea University Hospital, Rome, Italy; 23 Department of Radiological, Oncological and Anatomo-Pathological Sciences, University La Sapienza, Umberto I University Hospital, Rome, Italy; 24 Medical Oncology, Paolo Giaccone University Hospital, Palermo, Italy; 25 Department of Ematology & Oncology, Pugliese-Ciaccio Hospital, Catanzaro, Italy; 26 Medical Oncology Unit, Belcolle Hospital, Viterbo, Italy; 27 Medical Oncology Unit, Lanciano-Vasto, Chieti, Italy; 28 Medical Oncology Unit, Santa Maria Goretti Hospital, Latina, Italy; 29 Medical Oncology Unit, Fatebenefratelli Hospital, Rome, Italy; 30 Medical Oncology AO S. Croce and Carle Teaching Hospital, Cuneo, Italy; 31 Gynaecology – Oncology Unit, University Cattolica del Sacro Cuore, Rome, Italy; 32 Department of Medical Oncology, ASL Nuro, Nuoro, Italy; 33 Department of Medical Oncology, IRCCS Giovanni Paolo II, Bari, Italy; 34 Division of Medical Oncology, Pescara Hospital, Pescara, Italy; 35 Research Unit Phase I Trials and Oncology Unit, ASST, Monza, Italy; 36 Division of Medical Oncology, Reggio Calabria General Hospital, Reggio Calabria, Italy; 37 Department of Systems Medicine, Medical Oncology, University Tor Vergata, Rome, Italy; 38 Radiotherapy Department, IRCCS Regina Elena National Cancer Institute, Rome, Italy; 39 Biostatistics Unit, IRCCS Regina Elena National Cancer Institute, Rome, Italy; 40 Institute of General Pathology, University Cattolica del Sacro Cuore, Rome, Italy; 41 Department of Medical Oncology, IRCCS Foundation University A. Gemelli, Rome, Italy; 42 Sandro Pitigliani Medical Oncology Department, Hospital of Prato, Prato, Italy; 43 Scientific Direction, IRCCS Regina Elena National Cancer Institute, Rome, Italy

**Keywords:** HER2+ breast cancer, Trastuzumab/pertuzumab blockade, T-DM1 efficacy

## Abstract

**Background:**

HER2-targeting agents have dramatically changed the therapeutic landscape of HER2+ advanced breast cancer (ABC). Within a short time frame, the rapid introduction of new therapeutics has led to the approval of pertuzumab combined with trastuzumab and a taxane in first-line, and trastuzumab emtansine (T-DM1) in second-line. Thereby, evidence of T-DM1 efficacy following trastuzumab/pertuzumab combination is limited, with data from some retrospective reports suggesting lower activity. The purpose of the present study is to investigate T-DM1 efficacy in pertuzumab-pretreated and pertuzumab naïve HER2 positive ABC patients. We also aimed to provide evidence on the exposure to different drugs sequences including pertuzumab and T-DM1 in HER2 positive cell lines.

**Methods:**

The biology of HER2 was investigated in vitro through sequential exposure of resistant HER2 + breast cancer cell lines to trastuzumab, pertuzumab, and their combination. In vitro experiments were paralleled by the analysis of data from 555 HER2 + ABC patients treated with T-DM1 and evaluation of T-DM1 efficacy in the 371 patients who received it in second line. Survival estimates were graphically displayed in Kaplan Meier curves, compared by log rank test and, when possibile, confirmed in multivariate models.

**Results:**

We herein show evidence of lower activity of T-DM1 in two HER2+ breast cancer cell lines resistant to trastuzumab+pertuzumab, as compared to trastuzumab-resistant cells. Lower T-DM1 efficacy was associated with a marked reduction of HER2 expression on the cell membrane and its nuclear translocation. HER2 downregulation at the membrane level was confirmed in biopsies of four trastuzumab/pertuzumab-pretreated patients.

Among the 371 patients treated with second-line T-DM1, median overall survival (mOS) from diagnosis of advanced disease and median progression-free survival to second-line treatment (mPFS2) were 52 and 6 months in 177 patients who received trastuzumab/pertuzumab in first-line, and 74 and 10 months in 194 pertuzumab-naïve patients (*p* = 0.0006 and 0.03 for OS and PFS2, respectively).

**Conclusions:**

Our data support the hypothesis that the addition of pertuzumab to trastuzumab reduces the amount of available plasma membrane HER2 receptor, limiting the binding of T-DM1 in cancer cells. This may help interpret the less favorable outcomes of second-line T-DM1 in trastuzumab/pertuzumab pre-treated patients compared to their pertuzumab-naïve counterpart.

**Supplementary Information:**

The online version contains supplementary material available at 10.1186/s13046-020-01797-3.

## Background

Human epidermal growth factor receptor 2 (HER2) is a member of the HER family of receptor tyrosine kinases, also including the epidermal growth factor receptor (EGFR), HER3, and HER4. Ligands binding to EGFR, HER3 and HER4 induce homo- and heterodimerization among the family members. Despite the lack of specific ligands, activated HER2 homodimerizes in HER2 positive (HER2+) breast cancer (BC) cells and is then recruited as a preferred partner in heterodimers, resulting in the activation of cancer-driving pathways [1].

HER2 overexpression and/or gene amplification occurs in approximately 15–20% of BC, and is associated with a more aggressive behavior, with high rates of cell proliferation and metastasis, and poor patient outcomes [2]. HER2+ advanced breast cancer (ABC) has significantly benefited from the approval of several HER2-targeting agents in the last decades. Trastuzumab, a monoclonal antibody targeting HER2, has revolutionized the therapeutic landscape of HER2+ ABC [3]. Despite this, up to 40% of ABC patients show innate trastuzumab-resistance, and most patients develop acquired resistance whithin the first year of trastuzumab treatment [4, 5]. The approval of three additional anti-HER2 agents, i.e., lapatinib, pertuzumab, and trastuzumab emtansine (T-DM1), has converted HER2+ ABC into a highly treatable disease, with more favorable outcomes [6–9]. Pertuzumab is a monoclonal antibody binding HER2 at a different site compared with trastuzumab. A more comprehensive signaling blockade underlies the noticebly enhanced antitumor activity of trastuzumab and pertuzumab combination treatment [10]. Results from the CLEOPATRA trial showed an unprecedented median overall survival (mOS) advantage of 15.7 months in the pertuzumab arm, and the double-block combination has therefore become the new standard first-line treatment in HER2+ ABC [7]. T-DM1 is an antibody-drug conjugate of trastuzumab with emtansine (DM1), an antimicrotubule maytansine derivative [11, 12]. The activity of T-DM1 depends on both trastuzumab antitumor effects and intracellular DM1. Following T-DM1 binding to membrane HER2 receptor, the HER2-T-DM1 complex enters into the cell via receptor-mediated endocytosis [13]. Subsequently to the release from the lysosome, DM1-containing metabolites inhibit microtubule assembly, causing cell death [14].

In second-line treatment, after treatment with taxane and trastuzumab or as first-line in patients with rapid progression after adjuvant trastuzumab (≤6 months), TDM-1 has shown greater efficacy than lapatinib and capecitabine in the phase III EMILIA trial [8]. Consequently, TDM-1 has become the standard second-line treatment in HER2+ ABC patients.

To the aims of the present study, it is noteworthy that patients accrued in randomized trials of T-DM1 had not received prior pertuzumab. Therefore, we lack solid evidence on T-DM1 efficacy following trastuzumab/pertuzumab-containing regimens. Data from observational trials are limited. We have previously shown evidence of lower T-DM1 efficacy in trastuzumab/pertuzumab-pretreated patients providing data from a retrospective, multicentric study of 250 HER2+ ABC patients [15]. In a further retrospective evaluation of T-DM1 activity as second-line or later treatment from Dzimitrowicz and colleagues [16], results in terms of tumor response rates and progression free survival (PFS) appeared less favorable than those reported in randomized trials of T-DM1.

These findings suggest the need to investigate the biology of HER2 through sequential treatments in order to define the molecular basis for the appropriate therapeutic approach.

Based on the above reported evidence, we explored the effects of the exposure to trastuzumab and/or pertuzumab on HER2 receptor expression and cellular localization in HER2+ BC cell lines, and their effects on T-DM1 activity. The pre-clinical experiments were paralleled by the conduct of a large, multicentric, retrospective observational study, i.e., the SePHER study, Administration Sequence in Pertuzumab-pretreated HER2 + ABC patients, aimed to explore the efficacy of T-DM1 in light of prior trastuzumab/pertuzumab treatment in the real-world setting.

## Materials and methods

### Study approval

The SePHER study is a multicenter, observational trial with retrospective design including HER2+ ABC patients from 45 Italian cancer centers. This study was approved by the Institutional Review Board (IRB) of the Regina Elena National Cancer Institute, Rome, Italy [reference number: RS793/16(1815)]. The approval of the coordinating centre was tempestively notified to all the participating partners for further consideration and approval by the respective IRBs. An ad hoc written informed consent was developed and implemented for this study participants.

### Patients’ selection

Information on demographics, clinical, histopathological and immunohistochemical (IHC) features, anti-tumoral therapies and related outcomes were retrieved from patients’ medical records by specifically trained research assistants. All included patients were treated for advanced disease. Each patient was evaluated during treatment according to the follow-up strategies of each center. Clinical response was evaluated by response evaluation criteria in solid tumours (RECIST) criteria, version 1.1. Anonymized data were entered into a dedicated database with a SPSS operating interface. Median follow up was calculated starting from diagnosis of metastatic disease to death or date at last follow up. Endpoints for efficacy outcome included progression free survival (PFS) and overall survival (OS). Progression free survival for any specific line of treatment was calculated from the time of treatment start to the time of disease progression, interruption of treatment for toxicity, death or loss to follow-up. Overall survival was calculated starting from diagnosis of metastatic disease to death or last follow-up. Median PFS (mPFS) and OS (mOS) were calculated using the Kaplan-Meier product limit estimator method.

We first analyzed 371 patients treated with T-DM1 in second-line. We then split the whole cohort of 555 patients into five subgroups, as follows: 1. Patients treated in first-line without pertuzumab/second-line with T-DM1 (Number of patients, N, 194 patients); 2. Patients treated in first-line without pertuzumab/T-DM1 in third-line or beyond (N: 148); 3. Patients treated in first-line with pertuzumab/second-line with T-DM1 (N: 177); 4. Patients treated in first-line with pertuzumab/T-DM1 in third-line or beyond (N: 11); 5. Patients treated in first-line with T-DM1 (N: 25). The groups defined upon treatments’ sequence, as previously specified, were selected by hypothesizing a possible effect of the administration sequence on the main clinical outcomes, i.e., OS and PFS to first-line, second-line and third-line of treatment (PFS1, PFS2 and PFS3, respectively). Survival estimates were first computed for the whole cohort and then by treatments’ sequence. Subsequently, data on OS and PFS2 were also analyzed across strata defined by IHC tumor features, namely, estrogen receptor (ER) and progesterone receptor (PgR) positive (triple positive, TP), ER or PgR positive, and both hormonal receptors (HRs) negative.

Pathology assessment was performed in surgical specimens of primary tumors by dedicated pathologists at the participating centers as per national standards. Estrogen receptor and PgR status were determined at each center by IHC according to the local standards. Positivity was considered at a cut-off of ≥1%. HER2 testing was performed according to the 2013 ASCO/CAP guidelines on HER2 Testing in Breast Cancer. A positive HER2 status required an IHC score of 3+ or positive fluorescence in situ hybridization/cromogenic in situ hybridization (FISH/CISH).

### Generation of drug-resistant cell lines

Drug-resistant cells were obtained by continuous exposure of HER2+ BT474 and SkBr3 cell lines, obtained from the American Type Culture Collection (ATCC), to 20 μg/ml trastuzumab or pertuzumab, or 10 μg/ml trastuzumab + 10 μg/ml pertuzumab, for 2 months, followed by 7 months of culture in medium supplemented with 50 μg/ml trastuzumab or pertuzumab, or 25 μg/ml trastuzumab + 25 μg/ml pertuzumab. Further details are reported in the Supplementary materials and methods and are available online.

### Statistical analysis

Within the overall cohort of the 555 patients, the associations of interest were also evaluated in light of: a. T-DM1 administration in second- or subsequent line, and b. molecular subgroups, with these latter being set based on the results of IHC analysis and according to the criteria fully reported in the patients’ selection paragraph. The covariates used in the Cox uni/multivariate models included the following categorical variables: “first-line pertuzumab” (yes vs. no) and “treatment sequence”, which both concurred to define the five categories described in detail in the methods section; age; IHC subtype; Ki-67 (> 20% vs ≤20%); metastasis at diagnosis (yes vs no); number of metastatic sites (> 1 vs 1); visceral metastasis (yes vs no); brain metastasis (yes vs no); bone-only metastasis (yes vs no), lenght of PFS1 and disease free interval (DFI). This latter was calculated from the time of surgery to the time of metastatic disease diagnosis. Variables testing significant in univariate analyses were further tested in multivariate models. The level of statistical significance was set at *p* ≤ 0.05, with a 95% confidence interval (95%CI). The SPSS software (SPSS version 21.0, SPSS Inc., Chicago, IL) was used for all statistical evaluations.

Regarding the experiments in cell lines, all data were reported as mean +/− standard deviation. Differences were considered statistically significant when p ≤ 0.05, with a 95%CI. Student’s t test was performed for the comparison of results from all different tests (*p < 0.05, **p < 0.001, ***p < 0.0001).

## Results

### Generation of trastuzumab, pertuzumab, and trastuzumab+pertuzumab resistant SkBr3 and BT474 cells in vitro

**Fig. 1:**
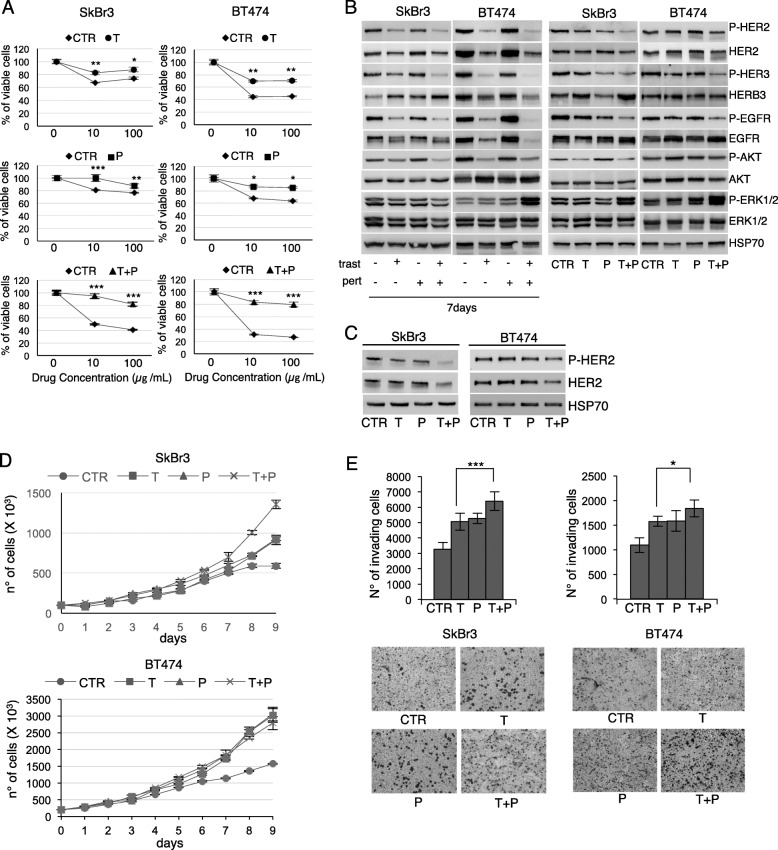
Characterization of resistant cell lines. Cell viability of control (CTR), Trastuzumab (T), Pertuzumab (P), and T + P cell lines treated with 10 and 100 μg/ml trastuzumab, pertuzumab and trastuzumab+pertuzumab for 5 days was evaluated by MTT assay (**a**). Western Blot (WB) analysis of phosphorylated and total HER2, HER3, EGFR, AKT and ERKs was performed on total cell lysates from untreated/treated parental cell lines for 7 days (**b**, leftmost panel). The basal levels of phosphorylated and total HER2, HER3, EGFR, AKT and ERKs were evaluated by WB in CTR, T, P, and T + P resistant cell lines (**b**, rightmost panel). HER2 total and phosphorylated levels evaluation by WB was repeated in CTR, T, P, and T + P cell lines following resistant cell lines establishment (**c**). The anti-HSP70 antibody was used to validate equivalent amount of loaded proteins in each lane. Proliferation curves (**d**) and Invasion assay (**e**, upper panel) of CTR, T, P, and T + P cell lines. Representative images of invaded cells are reported for each cell line (**e**, lower panel)

To investigate the mechanisms underlying lower T-DM1 efficacy following trastuzumab/pertuzumab in ABC patients, we established trastuzumab (T), pertuzumab (P), and trastuzumab+pertuzumab (T + P)-resistant HER2+ SkBr3 and BT474 cell lines (Fig. 1a). In agreement with previously reported data, Western Blot (WB) analysis revealed that short-term trastuzumab treatment inhibits the phosphorylation of HER family members and AKT, but not of ERKs (Fig. 1b, leftpanels) [17]. This effects were emphasized in trastuzmab+pertuzumab short term -treated cells. Wester Blot analysis of the generated resistant cell lines revealed reduced HER2 activity and expression and marked induction of ERKs phosphorylation in T + P cells as compared to parental and both T and P cells, whereas HER3 and EGFR were upregulated despite the reduced phosphorylation, in both BT474 and SkBr3 (Fig. 1b, right most panels). T + P cells maintained decreased HER2 phosphorylation and total expression, as confirmed by periodically testing resistant and control cell lines following their establishment (Fig. 1c). Resistant cell lines exhibited a higher proliferation rate (Fig. 1d) and invasive capability (Fig. 1e) compared to control cells. The number of invading T + P cells was significantly higher compared to T cells in both cell lines. Overall this data indicate that a more aggressive behavior is induced by the chronical exposure of HER2+ breast cancer cell lines to the combination trastuzumab/pertuzumab rather than trastuzumab.

### Dual HER2 blockade is associated with reduced T-DM1 efficacy due to HER2 downregulation

**Fig. 2:**
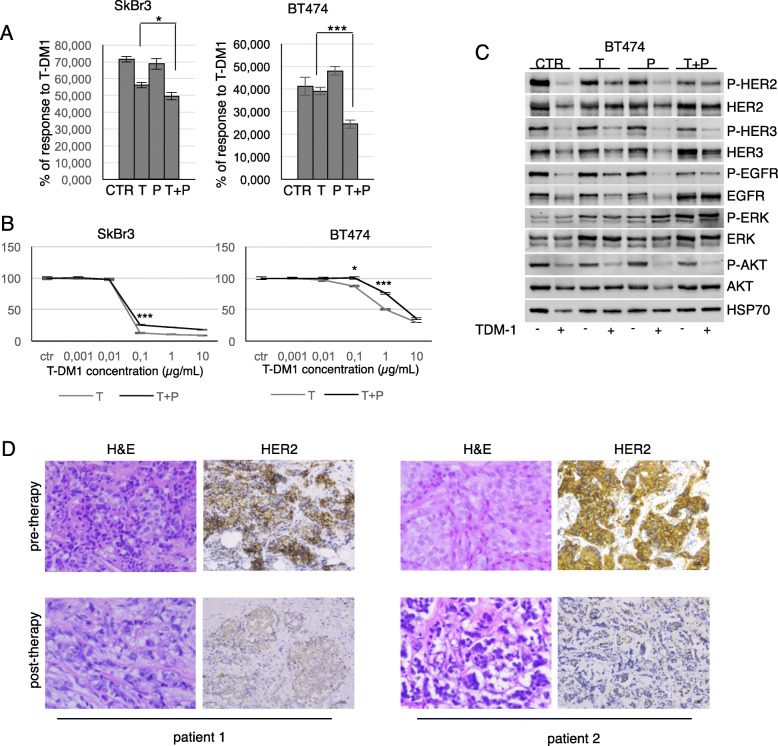
Trastuzumab+pertuzumab combination is associated with HER2 downregulation. Cell viability of CTR, T, P, and T + P SkBr3 and BT474 cells treated with 0.1 μg/ml and 1 μg/ml T-DM1, respectively, for 48 h was evaluated by Crystal Violet Assay (**a**). The results are expressed as percentage of T-DM1-responsive cells relative to control cells, as mean +/− standard deviation. T-DM1 dose-response curves of T and T + P cells are reported for both SkBr3 and BT474 (**b**). The expression of phosphorylated and total HER2, HER3, EGFR, AKT and ERKs was evaluated by Western Blot (WB) following exposure to 1 μg/ml T-DM1 for 48 h (**c**). Representative pre- and post-therapy sections from 2 ABC patients, stained by immunohistochemistry with eosin-hematossilin and anti-HER2 antibody (**d**)

Following T-DM1 treatment, the percentage of responsive T + P cells was significantly lower compared to T cells in BT474 (*p* < 0.0001), and in SkBr3, although at a lower level of statistical significance (*p* < 0.05) (Fig. 2a). Full T-DM1 dose-response curves of T and T + P cell lines are reported in Fig. 2b, that confirm significantly lower T-DM1 efficacy in cells chronically exposed to the combination trastuzumab/pertuzumab. T-DM1 induced a marked down-regulation of HER2 as well as HER3 and EGFR, in parental, T and P BT474 cells, whereas T + P BT474 cells maintained unmodified levels of these receptors (Fig. 2c). T-DM1 inhibited ERKs phosphorylation in control cells, T and P cells, whereas it considerably induced ERKs activation in T + P cells, in both cell lines (Fig. 2c, data available upon request). The downregulation of HER2 induced by trastuzumab/pertuzumab combination was confirmed in vivo, by the immunohistochemical assessment of bioptic specimens from four trastuzumab/pertuzumab-treated ABC patients prior to and following exposure to double-block (Table 1). Representative images of eosin-hematossilin and HER2 staining from two patients are shown in Fig. 2d.

**Table 1:** Immunohistochemical expression of Estrogen Receptor (ER), Progesterone Receptor (PgR) and HER2 in pre- and post-treatment bioptical tissue samples from 4 HER2+, pertuzumab pretreated, advanced breast cancer patients

	***Pre-treatment***					***Post-treatment***				
	ER	PgR	HER2 score	HER2%	FISH	ER	PgR	HER2 score	HER2%	FISH
*Patient 1*	90%	90%	2+	70%	amplified	90%	90%	0	0%	–
*Patient 2*	90%	40%	2+	30%	amplified	90%	60%	1+	30%	–
*Patient 3*	46%	0%	2+	100%	amplified	75%	18%	1+	15%	amplified
*Patient 4*	88%	42%	3+	100%	–	99%	1%	1+	60%	amplified

### The combination trastuzumab+pertuzumab induces HER2 nuclear translocation

**Fig. 3:**
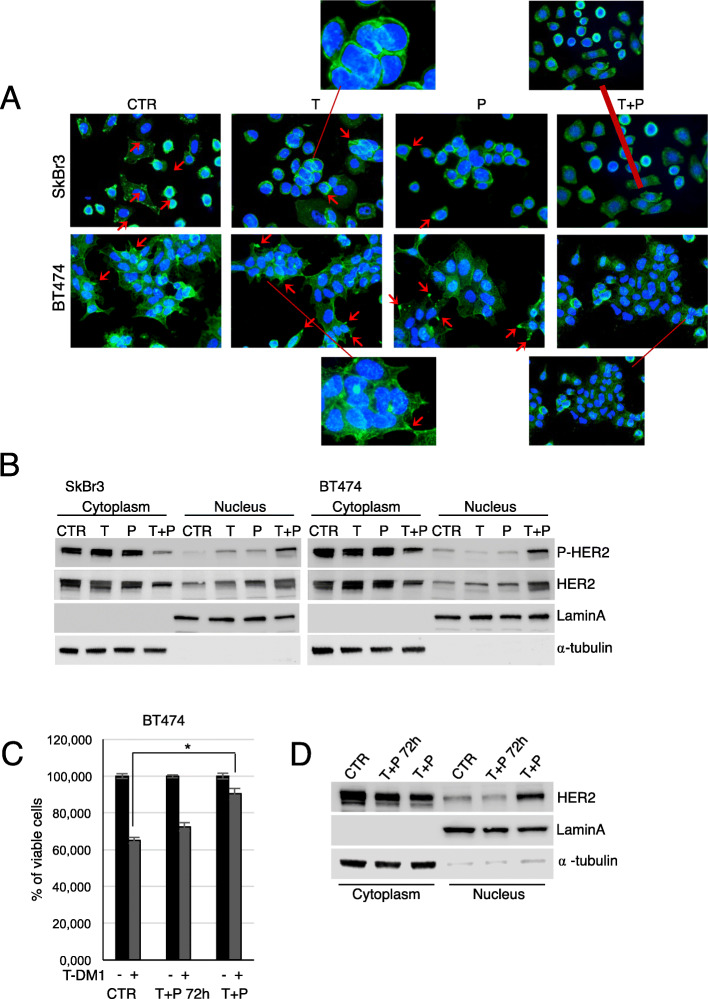
Prolonged trastuzumab+pertuzumab induces HER2 nuclear translocation. Control, T, P, and T + P cell lines were plated on poly-l lysine coated slides, and stained 24 hous later with anti-HER2 (green signal) (**a**). These cells were counterstained with Hoechst to highlight nuclei. Red arrows indicate HER2 localization on cellular protrusions. Cytoplasmic and nuclear fractions extracted from control, T, P, and T + P cells were analysed by Western Blot (WB) for the expression of phosphorylated and total HER2. Lamin A and α-tubulin were used to validate purity of nuclear and cytoplasmic extracts respectively (**b**). Following pre-treatment with 5 μg/ml trastuzumab + 5 μg/ml pertuzumab for 72 h, cell viability of control cells, pre-treated and T + P BT474 exposed to1 μg/ml T-DM1 for 72 h was evaluated by Crystal Violet Assay (**c**). Cytoplasmicand nuclear fractions of control, pre-treated and T + P BT474 cells were analysed by WB for the expression of total HER2 (**d**)

When administered in vitro as short-term treatments, trastuzumab and pertuzumab alone barely down-regulate HER2 [18–20], while their combination induces a stronger HER2 downregulation [19, 20]. To investigate the long-term effects of trastuzumab and pertuzumab on HER2 subcellular distribution, we performed immunofluorescence experiments. As shown in Fig. 3a, in control cells, T and PBT474 and SkBr3 cells, HER2 was mainly localized at the plasma membrane level, and concentrated on cellular protrusions [17]. A diffuse cytoplasmic signal was also present. Conversely, T + P cells lost membrane-HER2 and retained HER2 cytoplasmic distribution (Fig. 3a). Western Blot analysis of the cytoplasmic and nuclear fractions of control and resistant cells showed a marked translocation of HER2 to the nucleus in T + P BT474 and SkBr3 cells (Fig. 3b). Nuclear HER2 was phosphorylated, suggesting its active involvement in transcriptional control mechanisms [21, 22]. A short trastuzumab/pertuzumab pre-exposure of parental BT474 did not affect T-DM1 efficacy compared to control cells, and no HER2 nuclear translocation was observed (Fig. 3c and d). Hence, prolonged exposure to trastuzumab/pertuzumab induces the loss of membrane HER2 and its nuclear traslocation, that represent a mechanism of acquired resistance, which in turn reduces the T-DM1 targeting potential and efficacy.

### Patients’ cohort description

Overall, 555 patients were treated with T-DM1 for ABC. Table 2 shows the main patient- and tumor-related characteristics. Briefly, 25 patients (4.5%) received T-DM1 in first-line, following relapse while on/within 6 months from neo−/adjuvant treatment, 371 (66.8%) were treated in second-line, 96 (17.3%) in third-line, and 63 (11.4%) beyond the third-line. Details on treatments received in first-, second- and third-line for the whole population are available in Supplementary Table 1. The median length of follow-up for the whole population of 555 patients was 41 months.

**Table 2:** Clinicopathological characteristics of the study participants (*N* = 555)

Characteristics	N(%)
**Age yr**, median (range)	54 (26–87)
**Estrogen Receptor**	
*Negative*	202 (36.4)
*Positive*	353 (63.6)
**Progesterone Receptor**	
*Negative*	297 (53.5)
*Positive*	258 (46.5)
**Ki-67**	
*≤ 20*	103 (18.6)
*> 20*	389 (70.1)
*unknown*	63 (11.3)
**Grading**	
*G1*	6 (1.1)
*G2*	148 (26.7)
*G3*	339 (61.1)
*unknown*	62 (11.2)
**Immunohistochemical Subtype**	
*TP*	244 (44.0)
*ER or PgR positive*	109 (19.6)
*ER and PgR negative*	202 (36.4)
**Metastatic at Diagnosis**	
*No*	398 (71.7)
*Yes*	157 (28.3)
**Neo−/adjuvant treatment**^a^	
*Yes*	363 (91.2)
*No*	35 (8.8)
**Neo−/adjuvant trastuzumab**^a^	
*Yes*	212 (53.3)
*No*	186 (46.7)
**Metastatic Sites**	
*Visceral*	397 (71.5)
*Bone-Only*	25 (4.5)
*Brain*	155 (27.9)
**Number of Metastatic Sites**	
*1*	397 (71.5)
*2*	85 (15.3)
*> 2*	73 (13.2)
**Disease Free Interval in months, median**	
*No first-line Pertuzumab / T-DM1 in second-line*	40
*No first-line Pertuzumab / T-DM1 subsequent lines*	53
*First-line Pertuzumab / T-DM1 in second-line*	47
*No first-line Pertuzumab / T-DM1 in subsequent lines*	28
*T-DM1 in first-line*	17
**T-DM1 treatment line**	
*First-line*	25 (4.5)
*Second-line*	371 (66.8%)
*Third-line*	96 (17.3%)
*Subsequent lines*	63 (11.4%)

^a^For patients with early disease at diagnosis (398 patients)

Abbreviations: *N* Number; *yr* Years; *TP* Triple positive; *ER* Estrogen receptor; *PgR* Progesterone receptor

### Comparing clinical outcomes according to the T-DM1 line of treatment

**Fig. 4:**
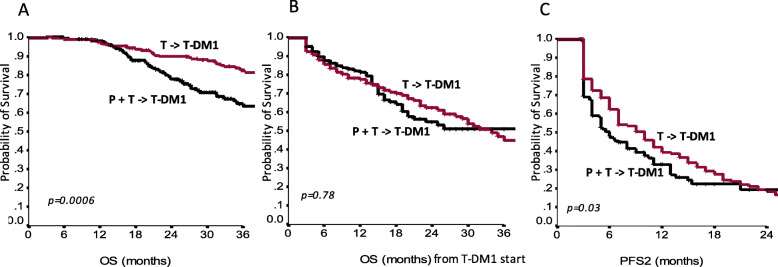
Overall survival (OS) from diagnosis of metastatic disease (**a**), OS from T-DM1 start (**b**) and progression free survival to the second-line of treatment (PFS2) (**c**), in patients treated with trastuzumab-based first-line and T-DM1 in second-line (T - > T-DM1) and in patients treated with pertuzumab-trastuzumab-based first-line and T-DM1 in second-line (P + T - > T-DM1)

Among the 371 patients who received T-DM1 in second-line, 177 had been treated with a trastuzumab/pertuzumab-based first-line, while the remaining 194 were pertuzumab naïve and had received a first-line trastuzumab-based regimen. In Table 3, we show the main baseline characteristics of pertuzumab naïve and pertuzumab-pretreated patients who received T-DM1 in second-line. Median OS from diagnosis of metastatic disease was 52 months in the pertuzumab pre-treated population vs 74 months in the pertuzumab naïve patients (*p* = 0.0006; Fig. 4a). Median OS calculated from the start of T-DM1 treatment was not significantly different, being not reached in the first group, and being 34 months in the second group (*p* = 0.78; Fig. 4b). Median PFS on first-line treatment (mPFS1) for patients who received a pertuzumab-trastuzumab-based regimen compared to those who received a trastuzumab-based regimen were 11 and 10 months, respectively (*p* = 0.22). Median PFS on second-line T-DM1 (mPFS2) was 6 months in patients who had received pertuzumab-trastuzumab in first-line compared to 10 months in those who were pertuzumab naïve (*p* = 0.03; Fig. 4c). Cox regression was performed for significantly differing outcomes between these two groups, namely OS and PFS2. Univariate analysis confirmed a negative impact of first-line pertuzumab for both OS [Hazard Ratio, HR 1.79; 95%CI 1.23–2.61; *p* = 0.002] and PFS2 [HR, 1.23; 95% CI 1.00–1.67; *p* = 0.049], which was exclusively confirmed in multivariate analysis of OS [HR 1.89; 95%CI 1.24–2.89; *p* = 0.003]. Conversely, among 159 patients who received T-DM1 in third-line or beyond, no differences by pertuzumab-pretreatment emerged for OS, PFS1 and PFS2 (adjusted *p*-values were 0.99, 0.76 and 0.26, respectively; Table 4).

**Table 3:** Clinical-pathological characteristics of the study participants who received T-DM1 in second-line after a trastuzumab-based first-line (*N* = 194) and after a pertuzumab-trastuzumab-based first-line (*N* = 177)

Characteristics	First-line trastuzumab/ Second-line T-DM1 [N (%)]	First-line pertuzumab-trastuzumab/Second-line T-DM1 [N (%)]	***p***-value
**Progesterone Receptor**			0.83
*Positive*	91 (46.9%)	85 (48.0%)	
*Negative*	103 (53.1%)	92 (52.0)%	
**Estrogen Receptor**			0.46
*Positive*	132 (68%)	114 (64.4%)	
*Negative*	62 (32%)	63 (35.6%)	
**Ki-67%**			**0.05**
*≤ 20*	45 (25.6%)	26 (16.6%)	
*> 20*	131 (74.4%)	131 (83.4%)	
**Immunohistochemical Subtype**			0.57
*TP*	87 (44.8%)	81 (45.8%)	
*ER or PgR positive*	45 (23.2%)	33 (18.6%)	
*ER and PgR negative*	62 (32.0%)	63 (35.6%)	
**Metastatic at Diagnosis**			**0.05**
*No*	147 (75.8%)	117 (66.1%)	
*Yes*	47 (24.2%)	60 (33.9%)	
**Number of Metastatic Sites**			0.14
*1*	142 (73.2%)	117 (66.1%)	
*> 1*	52 (26.8%)	60 (33.9%)	
Neo−/adjuvant treatment *			0.38
*Yes*	128 (66.0%)	109 (61.6%)	
*No*	66 (34.0%)	68 (38.4%)	
Neo−/adjuvant trastuzumab *			0.48
*Yes*	76 (39.2%)	63 (35.6%)	
*No*	118 (60.8%)	76 (39.2%)	
**Visceral Metastatic Site(s)**			0.11
*Yes*	133 (68.9%)	135 (76.3%)	
*No*	60 (31.1%)	42 (23.7%)	
**Disease Free Interval**^a^			0.10
*< 3 years*	66 (46.8%)	47 (41.2%)	
*≥ 3 years*	75 (53.2%)	67 (58.8%)	

^a^For patients with early disease at diagnosis

Abbreviations: *N* Number; *TP* Triple positive; *ER* Estrogen receptor; *PgR* Progesterone receptor

**Table 4:**
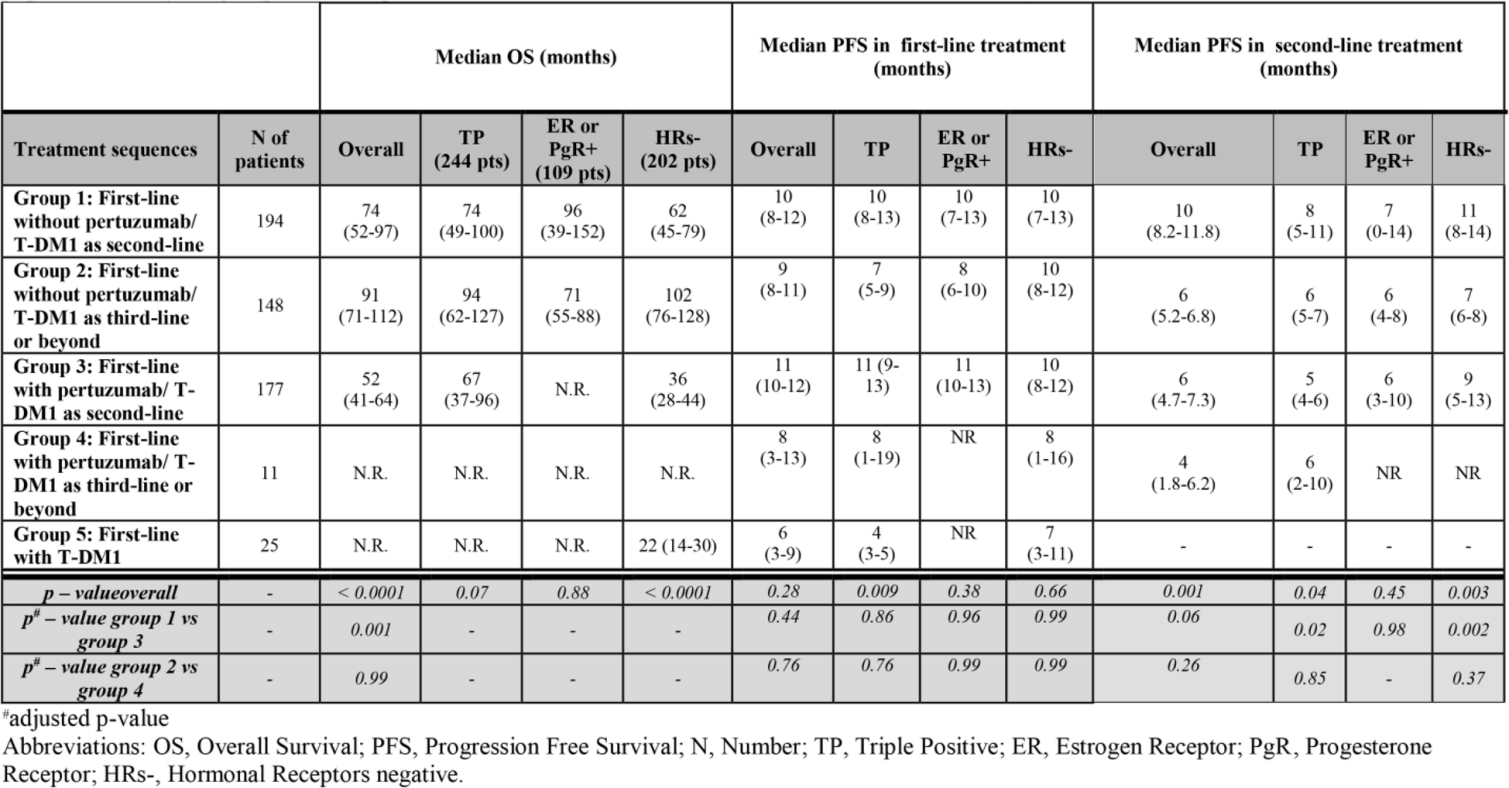
Median OS, median PFS in first-line of treatment and median PFS in second-line of treatment by treatment sequences in the overall population and by subgroups defined upon immunohistochemical characterization of molecules features (N:5555)

### Comparing clinical outcomes according to the treatment sequences and immunohistochemical subtypes

Overall, with a median follow up of 41 months, mOS for the whole cohort of 555 patients treated with T-DM1 in any treatment lines was 73 months. Altogether, the administration sequence of anti-HER2 agents significantly affected mOS (*p* < 0.0001) and mPFS2 (*p* = 0.001) (Table 4), with poorer outcomes in the group of patients having received pertuzumab-based first-line treatment followed by second-line T-DM1. No differences by trastuzumab/pertuzumab-pretreatment emerged for mOS, mPFS1 and mPFS2 in patients who received T-DM1 in third-line or beyond (Table 4). 

When the overall cohort of 555 patients was stratified by IHC subtype, a lower mOS in pertuzumab pre-treated patients was confirmed only in HRs-negative patients (*p* < 0.0001), while a shorter mPFS2 was observed in both TP (*p* = 0.04) and HRs-negative patients (*p* = 0.003). However, multivariate analysis showed no effect of the IHC-defined subtype on mOS, mPFS1 or mPFS2.

## Discussion

Resistance to HER2 targeting agents is a challenging topic in BC. Several pre-clinical studies explored potential resistance mechanisms to T-DM1, involving reduction of lysosomal proteolytic activity [23], STAT3 activation [24], HER2 genomic amplification [25], and *sustained signaling from* neuregulin*β1* [26]. Moreover, previous evidence has shown that trastuzumab-mediated engagement of immune effector cells induces HER2 downregulation in BC cells by STAT1 activation [27]. To our knowledge, no previous study has specifically focused on the potential cross-resistance mechanisms between pertuzumab pre-treatment and T-DM1 at a molecular level, particularly if compared to previously documented cross-resistance mechanisms due to trastuzumab alone.

We herein show first time evidence that dual HER2 blockade by trastuzumab/pertuzumab is associated with a marked inhibition of HER2 receptor expression on plasma membrane of cancer cells in vitro and in vivo, which limits the targetable HER2 receptor available to T-DM1. Our results also indicate that a significant amount of HER2 is translocated to the nuclei of trastuzumab+pertuzumab-resistant BC cells in vitro. Conversely, this effect is not shown in trastuzumab-pretreated BC cell lines. In agreement, our data from BC cell lines indicate that T-DM1 treatment induces internalization of membrane HER2 receptor in trastuzumab-resistant and pertuzumab-resistant BC cells lines; on the contrary, the expression levels of HER2 are not affected by T-DM1 in trastuzumab+pertuzumab-resistant BC cells, since the amount of membrane HER2 is already reduced by the previous double-block determining HER2 nuclear localization.

Nuclear HER2 has been reported to act as a transcriptional regulator and represents an independent prognostic factor of poor clinical outcome [21, 22]. In preclinical trastuzumab-resistant BC models, the inhibition of nuclear HER2 suppresses cell growth, indicating that nuclear HER2 is the major proliferation driver in trastuzumab-resistant BC. In this context, the inability of trastuzumab to disrupt the neregulinβ1-induced assembly of a nuclear HER2/HER3/STAT3 transcriptional complex has been demonstrated [28]. The lack of HER2 nuclear translocation following exposure to trastuzumab or pertuzumab alone highlights the synergy emerging from the combination of these two drugs.

These findings, together with the increased invasiveness and marked activation of MAPK/ERK pathway of trastuzumab+pertuzumab-resistant BC cell lines, suggest that the administration of pertuzumab in combination with trastuzumab favors the selection of a more aggressive phenotype of HER2+ BC cells, as compared to trastuzumab alone.

We have a preliminary evidence of higher tumor growth in mice subcutaneously injected with T + P resistant cells, as compared to T cells. The validation of reduced T-DM1 efficacy following trastuzumab+pertuzumab in a mouse model would strengthen our results, and experiments aimed at this goal are ongoing.

The selection of patients to be included in the present study was conditional in terms of considering only those who received T-DM1 at some point during their clinical history for advanced BC, and the analysis focused on trastuzumab/pertuzumab or trastuzumab-based pretreatment and second-line T-DM1 efficacy.

Among patients treated with T-DM1 in second-line, mOS from the start of the first-line and mPFS2 resulted longer in patients who had received a trastuzumab-based first-line, with respect to the counterpart pretreated with a pertuzumab/trastuzumab-based-regimen. The mPFS2 advantage observed within the first patient group may be reconciled with a higher efficacy of T-DM1 in patients pre-treated with trastuzumab alone, compared to those having received the double-block. Conversely, when addressing the advantage in mOS from the start of the first-line in the pertuzumab-naïve group, the link to differences in T-DM1 efficacy based on the administration sequence is less immediate. Indeed, we observed no difference in mOS from the start of T-DM1 between trastuzumab-pretreated and pertuzumab/trastuzumab-pretreated patients. In patients who did not received pertuzumab in first-line but for whom TDM-1 was instead already available at further disease progression, a quite long time to disease progression may be hypothesized, which may itself reflect a less aggressive biologic behavior on behalf of cancer. In brief, for patients in the pertuzumab-naïve group, the longer mOS from the first-line treatment may be more closely related to a more indolent disease course due to intrinsic disease characteristics than to the sequence of anti-HER2 agents administration.

The issue of a possible decrease in T-DM1 efficacy if given immediately after the double pertuzumab-based HER2 double-block has not been exhaustively addressed in previous studies [8, 9]. The EMILIA [8] and TH3RESA [9] trials were the two pivotal randomized phase III clinical studies that brought T-DM1 as a standard of care in second-line or beyond for patients with HER2+ advanced BC that progressed to standard treatments. TH3RESA trial showed clinical advantage by using T-DM1 compared to treatment of choice by the clinician also in patients that had received lapatinib and capecitabine, while EMILIA trial showed superiority of T-DM1 in second-line even when compared head to head with the lapatinib plus capecitabine regimen. Unfortunately, none of the patients included in the EMILIA and TH3RESA trials had received pertuzumab before being treated with T-DM1. Data from prospective studies is lacking in this context. Evidence on the topic under debate comes from three recent retrospective studies showing lower response rate and shorter survival outcomes in patients treated with T-DM1 following pertuzumab administration [15, 16, 29] In a previous retrospective multicentric study from our group involving 250 pretreated HER2 positive BC patients we showed lower efficacy of T-DM1 in the pertuzumab-pretreated cohort in comparison with the trastuzumab pretreated group [15]. In the Dzimitrowicz paper, authors showed results that are congruent with our findings [16]. In this study, including only patients that had received a pertuzumab-trastuzumab-based first-line, a lower efficacy of T-DM1 in terms of response rate was reported when compared to the response rates observed in the randomized clinical trials, where patients were only trastuzumab resistant. In the Noda-Narita et al. retrospective study enrolling 42 advanced HER2 positive patients, median PFS and objective responses were lower in the group pretreated with pertuzumab/trastuzumab in comparison to the trastuzumab subgroup [29].

The main limitation of the observational section of the present study is its retrospective, multicenter design, which per se represents a considerable source of data heterogeneity. Moreover, the lack of central assessment on IHC features of primary and metastatic lesions deserves mentioning, although quality controls routinely performed at the pathology labs of the institutions involved increase our confidence in data quality. At the same time, the involvement of a relevant number of cancer centres/oncologic divisions, i.e., N: 45, has allowed to collect and analyze the largest amount of data ever made available to investigate the efficacy of T-DM1 following trastuzumab/pertuzumab-based treatment. Beyond its intrinsic limitations and bias, this approach allowed us to confirm the reduced T-DM1 efficacy following dual HER2 blockade by trastuzumab/pertuzumab in HER2+ ABC patients.

Our choice of relying on an observational study with a retrospective approach has paved the way to confounding and bias, which we attempted to minimize in the phase of data analysis throughout stratification and statistical modeling. In more detail, concerning stratification, data were analyzed within strata defined upon a given pre-specified variable. In doing so, we managed the confounding effects of a given variable possibly acting as a confounding at the price of a reduction of the study power in detecting the association of interest in the case of small strata. Within the SePHER study, statistical modeling translated into the development of multivariable Cox models. These latter approach allowed to simultaneously control for more than one confounder at the time, and help interpret the effect of each confounder in light of the others. In addition, data retrieving was performed by ad hoc trained research assistants who worked in strict collaboration with the oncologists involved at the single centre level. This may have minimized the chances of residual confounding.

The main strength of the present study is that, to our knowledge, we first reported on HER2 downregulation as a key mechanism underlying lower T-DM1 efficacy observed in the clinical setting when this drug is administered as second-line therapy in trastuzumab/pertuzumab-pretreated HER2+ ABC patients. Results from the experiments performed in bioptic specimens of trastuzumab/pertuzumab pretreated advanced breast cancer patients were further confirmative. Indeed, when comparing the pre- and post-treatment HER2 scores for each of the 4 patients examined, the IHC assessment uniformly showed a score reduction. This evidence provides support to our study hypothesis, in that the reduced HER2 scores at the IHC evaluation of the post-treatment samples are in key with a lower availability of the HER2 at the membrane level, which may per sè at least partly account for less favorable outcomes in patients exposed to pertuzumab.

As shown in Tables 1, 2 cases among the 4 assessed resulted negative at the FISH in the post-treatment window of evaluation. The co-existence of HER2-negative and HER2-positive clones is plausible from a biological standpoint. In addition, it may reflect the selective pressure applied by the prior administration of anti-HER2 agents. Indeed, HER2-negative clones may concur to less favorable outcomes in patients treated with anti-HER2 agents. The extent to which the “degree” of HER2-negative clone selection has been driven by the specific sequence of anti-HER2 agents administration and/or this mechanism concurs with the mechanism we have originally hypothesized deserves further investigation in more adequately sized samples.

Although hypothesis-generating, data from the study herein presented, as well as from prior similar studies within this same research pipeline, are limited in nature. Still, they provided an appropriate ground in terms of preliminary evidence, on which we designed a randomized clinical trial investigating the optimal treatment sequence in HER2-positive ABC patients. In more detail, our team at the IRCCS Regina Elena National Cancer Institute is the coordinating center of the STEP trial, an active randomized multicenter prospective trial exploring the optimal Sequence TrEatment in HER2+ Pertuzumab-pretreated ABC patients. The STEP trial was granted formal approval and financial support by the Italian Ministry of Health (project code: GR-2018-12,367,431). Evidencefrom the STEP and similar ad hoc, prospective randomized trials are eagerly awaited to delineate the optimal treatment sequence in HER2 + ABC patients, in order to gain more favorable treatment outcomes in this patients’ population.

## Conclusion

Overall, our findings suggested HER2 downregulation following dual HER2 blockade by trastuzumab/pertuzumab as a key mechanism underlying lower T-DM1 efficacy as second-line therapy in HER2+ ABC patients. Results from our retrospective study showed indeed lower T-DM1 efficacy in terms of mOS and mPFS in 177 patients who received trastuzumab/pertuzumab in first-line, as compared to 194 pertuzumab-naïve patients. In addition, we showed HER2 nuclear translocation in trastuzumab+pertuzumab-resistant HER2+ BC cell lines in vitro.

The design of prospective randomized trials may lead to delineate the optimal treatment sequence in HER2 + ABC, and ultimately to more favorable outcomes in these patients’ population.

## Supplementary Information

**Additional file 1.**

**Additional file 2.**

## Data Availability

The datasets used and/or analyzed during the current study are available from the corresponding author on reasonable request.

## Abbreviations

HER2: Human epidermal growth factor receptor 2
EGFR: Epidermal growth factor receptor
BC: Breast cancer
ABC: Advanced breast cancer
T-DM1: Trastuzumab emtansine
PFS: Progression free survival
OS: Overall survival
IHC: Immunohistochemistry
IRB: Institutional review board
RECIST: Response evaluation criteria in solid tumours
ER: Estrogen receptor
PgR: Progesterone receptor
HRs: Hormonal receptors
FISH: Fluorescence in situ hybridization
CISH: Cromogenic in situ hybridization
