# Supplementary material for: Loss of HER2 and decreased T-DM1 efficacy in HER2 positive advanced breast cancer treated with dual HER2 blockade: the SePHER Study
Source: J Exp Clin Cancer Res. 2020 Dec 10;39:279. doi: 10.1186/s13046-020-01797-3 (PMC7731769; doi:10.1186/s13046-020-01797-3)
Supplement: Supplementary file 1 — Additional file 1. [file 13046_2020_1797_MOESM1_ESM.docx]

**Supplementary Table 1.** Treatment administered in first and subsequent lines in the subset of study participants with available data on progression free survival to first-line therapy (N=555).

|  | **FIRST-LINE** | | **SECOND-LINE** | | **THIRD-LINE** | |
| --- | --- | --- | --- | --- | --- | --- |
|  | **N** | **% of patients who received 1^st^ line** | **N** | **% of patients who received 2^nd^line** | **N** | **% of patients who received 3^rd^ line** |
| **Pertuzumab-trastuzumab + CT** | 188 | 33.9% | 6 | 1.1% | 0 | 0% |
| **T-DM1** | 25 | 4.5% | 371 | 70.0% | 96 | 64.4% |
| **Trastuzumab+ CT** | 256 | 46.1% | 100 | 18.9% | 40 | 26.8% |
| **Trastuzumab+**  **ET** | 22 | 4.0% | 0 | 0% | 1 | 0.7% |
| **CT alone** | 46 | 8.3% | 18 | 3.4% | 13 | 8.7% |
| **ET alone** | 6 | 1.1% | 3 | 0.6% | 0 | 0% |
| **Lapatinib + Capecitabine** | 6 | 1.1% | 28 | 5.3% | 19 | 12.6% |
| **Trastuzumab alone** | 6 | 1.1% | 4 | 0.8% | 5 | 3.4% |
| **No therapy** | - | - | 25 | - | 381 | - |
| **Treatments administered** | 555 | 100% | 530 | 100% | 149 | 100% |

**Abbreviations:** N: Number; CT: chemotherapy; ET: endocrine therapy.
