# Supplementary material for: Loss of HER2 and decreased T-DM1 efficacy in HER2 positive advanced breast cancer treated with dual HER2 blockade: the SePHER Study
Source: J Exp Clin Cancer Res. 2020 Dec 10;39:279. doi: 10.1186/s13046-020-01797-3 (PMC7731769; doi:10.1186/s13046-020-01797-3)
Supplement: Supplementary file 2 — Additional file 2. [file 13046_2020_1797_MOESM2_ESM.docx]

**Materials and Methods**

**Cell lines and treatments**

The HER2+ breast cancer (BC) cell lines BT474 and SkBr3 were obtained from the American Type Culture Collection (ATCC) and maintained in RPMI and DMEM mediums, respectively, supplemented with 10% FCS, 1% penicillin/streptomycin and 1% glutamine (Invitrogen, Milan, Italy). The cell lines were periodically tested for mycoplasma contamination.

The anti-HER2 monoclonal antibodies trastuzumab and pertuzumab, and T-DM1 were purchased from the pharmacological service of our institute. For the 7 day-treatments of BT474 and SkBr3 cell lines, the following concentrations of drugs were used: trastuzumab and pertuzumab 10 μg/ml, trastuzumab 5μg/ml + pertuzumab 5 μg/ml. T-DM1 treatments of BT474 and SkBr3 cell lines were conducted for 48 hours at drug concentrations of 1 μg/ml (BT474) and 0,1 μg/ml (SkBr3), corresponding to IC50 values as estrapolated from dose-response curves.

**Generation of drug-resistant cell lines**

To obtain drug-resistant cell lines, BT474 and SkBr3 cells were cultured in complete medium supplemented with 20 μg/ml trastuzumab, 20 μg/ml pertuzumab, and 10μg/ml trastuzumab + 10μg/ml pertuzumab, for two months, followed by 7 months of culture in medium supplemented with 50 μg/ml trastuzumab, 50 μg/ml pertuzumab, and 25 μg/ml trastuzumab + 25 μg/ml pertuzumab. Following their establishment, resistant cell lines were continuously cultured in presence of drug/drugs; treatments were removed 72 hour prior to experiments.

**Cell growth and viability assay**

To check for drug-resistant cell lines obtained, resistant and control cell lines were seeded in 96-multiwell culture plates at density of 5x10^3^ cells/well and incubated overnight at 37 ̊C. Cells were then treated with 10 and 100 μg/ml trastuzumab, pertuzumab and their combination for 5 days. After incubation, MTT reagent (0.5 mg/ml) was added to each well, and the plates were incubated in the dark at 37 ̊C for 4h. At the end of the incubation, the medium was removed, the resulting formazan was dissolved in isopropanol, and the optical density was measured at 540 nm using an ELISA plate reader.

For T-DM1 dose-response curve, cells were seeded in 24-multiwell culture plates at density of 5x10^4^ cells/well and incubated overnight at 37 ̊C. Then, they were treated with different concentrations of T-DM1 (0.001; 0.01; 0.1; 1 and 10 μg/ml) for 72 hours. After incubation, the cells were fixed with 4% formaldehyde for 15 minutes and stained with 0.1% crystal violet for 40 minutes. The plates were washed with H2O and air-dryed, and the stain was extracted by 10% glacial acetic acid. Absorbance was read at 570 nm on a microplate reader.

**Antibodies**

Rabbit anti-phospho ser473 (#9271) and total AKT (#9272), anti-phospho (#9101L) and total ERKs 1/2 (#9102), anti-phospho tyr1248 (#2247) and total HER2 (#2165), anti-phospho tyr1068 (#3777) and total EGFR (#4267), anti-phospho tyr1289 (#4791) and total HER3 (#12708) antibodies were purchased from Cell Signaling (Milan, IT). Mouse anti-HSP70 (N27F34) antibody was purchased from Stressgen (Milan, IT). These antibodies were used in Western Blot experiments. Rabbit anti-HER2 (A0485) was purchased from Dako(Milan, IT), and used for Immunohistochemistry. Mouse anti-HER2 (MA5-13675) was purchased from Invitrogen (Milan, IT), and used in immunofluorescence experiments. Peroxidase-conjugated secondary antibodies anti-IgGs were purchased from Cappel and/or BioRad (Milan, IT). Alexa-Fluor 488 Donkey anti-mouse secondary antibody was from Invitrogen (Milan, IT).

**Western blot analysis**

To analyze phosphor- and total HER3, phosphor- and total HER2, phospho- and total EGFR, phosphor- and total ERKs 1/2, phospho- and total AKT and HSP70 proteins expression, the cells were lysed with RIPA buffer [50 mMTris (pH 8), 150 mMNaCl, 1% Nonidet P40, 0.1% deoxycholate, 0.1% SDS, 1 mM PMSF, 5 mM Na3VO4, 50 mM protease inhibitors (SIGMA-Aldrich, Milan, IT)] for 30 minutes at 4°C. Total cell lysates were clarified by centrifugation at 14,000 rpm for 30 minutes at 4°C. For isolation of cytoplasmic and nuclear fractions, NE-PER ^TM^ Nuclear and Cytoplasmic Extraction Reagents from Thermo SCIENTIFIC (Milan, IT) were used, according to the manufacturer guidelines. Aliquots of cell extracts containing equivalent amount of proteins were treated at 65°C for 5 minutes, resolved by SDS-polyacrilamide gel electrophoresis 8% (SDS-PAGE), and transferred to nitrocellulose. The horseradish peroxidase-conjugated goat anti-mouse or anti-rabbit were used as secondary antibodies. Signals were detected by LuminataTMClassico Western HRP substrate (Millipore). Same amount of total protein from three independent experiments were pooled and analyzed. Total loaded proteins were normalized by anti-HSP70.

**Cell proliferation**

Control and drug-resistant BT474 and SkBr3 cells (1×105) were plated onto 100 mm^2^ plates. Every 24 h cells were collected and counted in 0.2% trypan blue solution, whereas medium changes were performed for the remaining samples every 48 hours. Each assay was repeated at least three times.

**Cell migration and invasion assays**

Chemotaxis and chemoinvasion assays were performed with control and drug-resistant BT474 and SkBr3 cells. Chemotaxis and chemoinvasion assays were assessed using a 48-well modified Boyden’s chamber (NeuroProbe, Pleasanton, CA) and 8-mm pore polyvinylpyrrolidone–free polycarbonate Nucleopore filters (Costar, New York, USA). The lower compartment of the chamber was filled with conditioned serum free medium produced from NIH3T3 fibroblasts. The cells were placed in the upper compartment of the Boyden’s chamber. To perform chemoinvasion assay the filters were pre-coated with 20μg/ml Matrigel (BD Biosciences, Milan, Italy). After 6 or 8 h of incubation at 37 °C, migrated or invaded cells on the lower surface of the filters were fixed, stained with DiffQuick (Merz-Dade, Dudingen, Switzerland) and counted. Each assay was carried out in quadruplicate and repeated at least three times. The ability of the cells to adhere to the filters was verified by staining the upper side of the filter for each cell line. Images were obtained by Microscope OLYMPUS BX53; scale bars = 20 μm.

**Immunofluorescence**

Control (C), trastuzumab (T), pertuzumab (P), and trastuzumab+pertuzumab (T+P)-resistant BT474 and SkBr3 cells were plated on poly-l lysine coated slides. 24 hours later cells were rinsed with ice-cold PBS and fixed with 2% formaldehyde for 10 minutes, followed by permeabilization with 0,25% Triton X100. The cells were blocked with 3% BSA and stained with anti-~~ErbB2~~ HER2 (1:500) for 2 hours. The cells were then washed with ice-cold PBS three times for 5 minutes each, and incubated with Alexa Fluor 488-labeled anti-mouse (1:200) for 1 hour. The cells were counterstained with Hoechst to highlight nuclei (SIGMA-Aldrich). Microscope OLYMPUS BX53 was used to evaluate fluorescence.

**FISH Procedure**

FISH was performed using the ZytoLight^®^ SPEC CEN17/ERBB2 Dual Color Probe and the ZytoLight^®^ FISH-Tissue Implementation Kit (ZYTOVISION, Bremerhaven, Germania), which includes a mixture of an green fluorochrome direct labeled CEN 17 probe specific for the alpha satellite centromeric region of chromosome 17 (D17Z1) and a orange fluorochrome direct labeled SPEC ERBB2 probe specific for the chromosomal region 17q12-q21.1 harboring the ERBB2 gene.

The assay was performed according to the manufacturer’s instructions.

In brief, after deparaffinization, FFPE specimens, were incubated in the pretreatment solution (98°C, 15 minutes) and then digested with protease (37°C, 10 minutes). After washing, slides were counterstained with 4’,6-diamidino-2 phenylindole (DAPI) and analyzed under a fluorescence microscope.

An average of 100 nuclei was enumerated in the invasive component of tumor tissue. Samples with a ratio value ≥2.0 were considered to be amplified. The sections were analyzed using a fluorescence microscope (X100).

**Immunohistochemistry**

Formalin-fixed paraffin-embedded sections from 4 patients with HER2+ ABC surgically treated at the Regina Elena National Cancer Institute and at the Candiolo Cancer Institute were analyzed for the expression of HER2. More specifically, we analyzed: (i) specimens derived from HER2+ advanced BC patients before perrtuzumab-based treatment; (ii) specimens derived from the same HER2+ advanced BC patients after pertuzumab-based therapy. Three-micrometer sections of formalin-fixed paraffin-embedded breast cancer tissue samples were cut on SuperFrost Plus slides (Menzel-Gläser, Braunschweig, Germany), and incubated with anti-HER2 (1:200). Immunoreactions were revealed by a streptavidin-biotin enhanced immunoperoxidase technique (Super SensitiveMultiLink) in an automated autostainer (Bond III, Leica Biosystems). Diaminobenzidine was used as chromogenic substrate. The IHC levels of HER2 expression were scored semi-quantitatively based on staining intensity and distribution percentage using the immune-reactive score (IRS, staining intensity × percentage of positive cells).
